# Nontuberculous mycobacterial infection after lung transplantation: a report of four cases

**DOI:** 10.1186/s40792-019-0565-1

**Published:** 2019-01-23

**Authors:** Naoko Ose, Masato Minami, Soichiro Funaki, Takashi Kanou, Ryu Kanzaki, Yasushi Shintani

**Affiliations:** Department of General Thoracic Surgery, Osaka University Graduate School of Medicine, 2-2 Yamadaoka, Suita-shi, Osaka Japan

**Keywords:** Nontuberculous mycobacteriosis, NTM, Infection, Lung, Transplantation

## Abstract

**Background:**

Nontuberculous mycobacterium (NTM) infection in a patient in an immunosuppressed state caused by increased use of immunosuppressive or biological agents is a serious clinical problem. *Mycobacterium avium complex* is the most common involved pathogen, followed by *Mycobacterium abscessus* (MABSC), while *Mycobacterium kansasii* is not a major concern. The rate of infection rate in lung transplant recipients is reported to range from 1.5–22.4%.

**Case presentation:**

We report here four cases of NTM pulmonary infection and disease among 63 patients who underwent lung or heart-lung transplantation at our hospital. Those four occurred following living-donor transplantation in two patients, one with pulmonary arterial hypertension and one with bronchiectasia, and deceased donor lung transplantation in two patients, lymphangioleiomyomatosis and interstitial pneumonia, respectively. NTM was not detected in any of the patients prior to transplantation. The involved pathogens were *Mycobacterium gordonae* in one, MAC in one, and MABSC in two of these patients, which were isolated from broncho-alveolar lavage (BAL) in two and sputum in two. The one case of MAC and two of MABSC were symptomatic with consolidation shown in chest CT images indicating possible pneumonia, while the one with *M. gordonae* had no symptoms and was detected by surveillance BAL. Onset time from detection of NTM was greater than 3 years in the three with MABSC and *M. gordonae* and less than 3 years in the one with MAC. Each patient required a decrease in immunosuppressive agents according to their condition, while antibiotics therapy was performed in the three who were symptomatic. Sputum culture findings became negative after several months and were maintained thereafter in all.

**Conclusion:**

An NTM infection leading to pulmonary disease can occur at any time following lung transplantation. Treatment should be considered depending on the involved pathogens, individual status, and disease severity.

## Background

The number of patients with pulmonary nontuberculous mycobacterium (NTM) is increasing worldwide [1, 2], with NTM infection in patients in an immunosuppressed state caused by increased use of immunosuppressive or biological agents a serious clinical problem. Isolation of NTM organisms following lung transplantation is reported to occur in 1.5–22.4% [3–11] of those patients, some of whom suffer from pulmonary disease. The most common strain varies among regions and countries, which is an important factor because effective treatment methods differ depending on the causative pathogen. Furthermore, onset period and infection site in the native or transplanted lung are critical for effective treatment.

We report here four cases of NTM pulmonary infection in patients who underwent lung transplantation and were treated at our institution and also review previous reports with focus on onset time and NTM strain. Of 63 patients at our hospital who underwent lung transplantation between January 2000 and January 2018, including heart-lung and living-donor transplantation, four (6.3%) had NTM isolated from obtained specimens, while NTM was not detected in any prior to transplantation. Surveillance bronchoscopy examinations were performed four times the first year and then annually until 5 years after transplantation, according to our institutional protocol. After the fifth year, clinically indicated bronchoscopic examination is occasionally performed when rejection or respiratory infection suspects. In the present cases, clinical samples were obtained from sputum and bronchial lavage samples obtained with bronchoscopy. Mycobacterial cultures were performed using Ogawa medium or a Mycobacteria Growth Indicator Tube system. No *Mycobacterium (M.) tuberculosis* organisms were isolated in any of the present cases.

## Case presentations

### Case 1

**Fig. 1:**
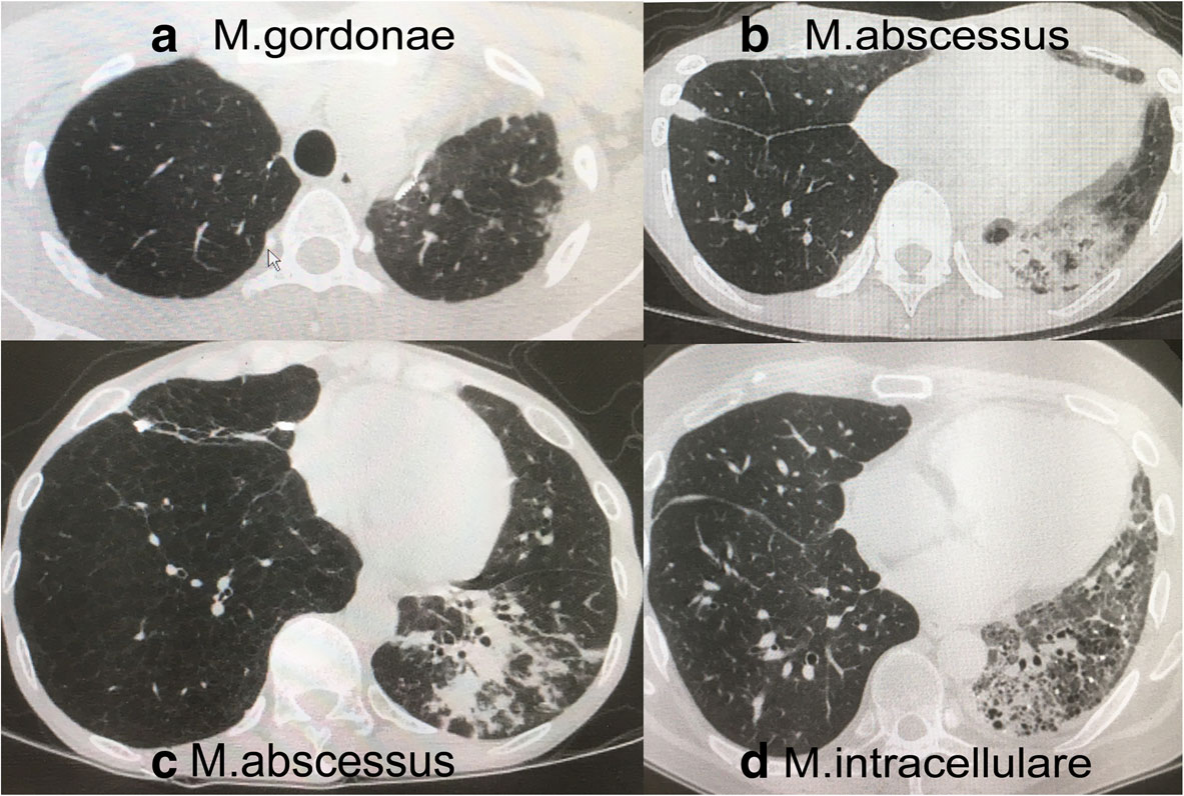
**a**–**d**. Chest computed tomography showing consolidation. **a** Case 1, slight consolidation in left upper lesion caused by *M. gordonae*. **b** Case 2, consolidation in left lower lesion caused by *M. abscessus*. **c** Case 3, consolidation in transplanted lung caused by *M. abscessus*. **d** Case 4, consolidation in native lung caused by *M. intracellulare*

An 11-year-old boy with pulmonary arterial hypertension underwent living-donor lung transplantation, with tacrolimus, mycophenolate mofetil (MMF), and prednisolone (PSL) given as immunosuppressive agents. At 76 months after transplantation, *M. gordonae* was isolated from a broncho-alveolar lavage (BAL) sample obtained during a surveillance examination. Contamination was suspected, because even though there were no symptoms, chest computed tomography (CT) showed a slight amount of consolidation in the left upper lesion (Fig. 1a). PSL as therapeutic and diagnostic treatment was decreased. Sputum culture findings were negative after 5 months, and chest CT images were clear. There was no further NTM detection during the following 15 years.

### Case 2

A 38-year-old female with bronchial ectasia underwent living-donor lung transplantation, with ciclosporin, MMF, and PSL given as immunosuppressive agents. At 82 months after transplantation, the patient developed a fever with purulent sputum and chest CT showed consolidation in the left lower lesion (Fig. 1b). *M. abscessus* complex (MABSC) was isolated from a BAL sample. Following administrations of tazobactam/piperacillin and azithromycin (AZM), as well as a decrease in ciclosporin from 120 to 50 mg for 1 month, the sputum cultures became negative. AZM administration and decreased ciclosporin were continued for 22 months, with no recurrence noted.

### Case 3

A 39-year-old female with lymphangioleiomyomatosis underwent single deceased donor lung transplantation, with ciclosporin, MMF, and PSL given as immunosuppressive agents. At 58 months after transplantation, a fever developed and chest CT showed consolidation in the transplanted lung (Fig. 1c). MABSC was isolated in a cultured sputum sample. Following administrations of imipenem, amikacin, and AZM for 4 months, sputum culture findings became negative. Maintenance therapy with imipenem and amikacin was given once a week, along with daily AZM and a decrease in MMF, with no recurrence seen during the following 1-year period.

### Case 4

A 41-year-old male with interstitial pneumonia underwent single deceased donor lung transplantation, with ciclosporin, MMF, and PSL given as immunosuppressive agents. At 12 months after transplantation, a fever developed and chest CT showed consolidation in the native lung (Fig. 1d), which was suspected to be pneumonia caused by general bacteria. Broad-spectrum antibiotic therapy was started, though was not effective. After a period of time, *M. intracellulare* was isolated from a cultured sputum sample. Thereafter, rifampicin (RFP), ethambutol, and clarithromycin (REC) treatment was administered for 3 months, after which sputum findings were negative. Rifampicin was continued at 800 mg, the standard level, while ciclosporin was adjusted according to trough level and finally administered at 550–600 mg, three times the normal dose, and PSL was gradually reduced. The patient died after 21 months because of respiratory failure due to chronic rejection, though sputum culture findings remained negative with REC treatment These cases are summarized in Table 1.

**Table 1:** Summary of 4 cases

Case No.	Age	Sex	Primary disease	Donar	Procedure	Period to onset^a^ (Month)	Organism	Infection lung	Symptom	Diagnosis	Drug	Period to negative conversion (month)	Treatment period (month)	Reinfection	outcome
1	10	M	PAH	Living	Bilateral	Late (76)	M.gordonae	Transplanted	None	BAL	None	5	–	None	Alive
2	35	F	BE	Living	Bilateral	Late (82)	MABSC	Transplanted	Fever Sputum	BAL	TAZ/PIPC+AZM	1	22	None	Alive
3	41	F	LAM	Cadaveric	Single	Late (58)	MABSC	Transplanted	Sputum	Sptum	AZM + IPM + AMK	4	12	None	Alive
4	39	M	NSIP	Cadaveric	Single	Early (12)	M.intracellurare	Native	Fever	Sptum	REC	3	21	None	Dead

*AZM* azithromycin, *BAL* broncho-alveolar lavage, *BE* bronchiectasis, *CyA* Cycrosporin, *LAM* lymphangioleiomyomatosis, *M.* Mycobacterium, *MMF* mycophenolate mofetil, *NSIP* nonspecific interstitial puemonia, *PAH* pulmonary arterial hypertension, *PSL* prednisolone, *REC* Rifampicin, ethambutol, and clarithromycin

^a^Early onset considered to occur within 180 days, late onset from 180 days until 3 years, and very late onset after 3 years

## Discussion

The number of patients worldwide with pulmonary NTM disease is increasing [1, 2]. An NTM infection is not contagious, whereas an MABSC infection can be acquired through transmission, potentially via fomites or aerosols [12]. Floto et al. reported that an NTM infection occurring in patients with cystic fibrosis can be problematic, as that can influence the indication for lung transplantation [13].

NTM infection following transplantation is a critical issue, because treatment is difficult, especially for patients in an immunosuppressed condition, as there is no specific medicine available for NTM, unlike *M. tuberculosis*. In hematopoietic stem cell or other solid organ recipients, the most common site of infection is the lung, with *M. avium* complex (MAC), the most commonly noted causative pathogen, followed by *M. kansasii* [14, 15]. Other studies have suggested that NTM infection rates of incidence in lung transplant recipients range from 1.5–22.4% (Table 2) [3–11]. In those reports, MAC was most common, followed by MABSC, while *M. kansasii* was not a major concern. However, there are also reports of rare types of NTM (Table 3), though the causative pathogens vary depending on region. In those cases, the most common infection site is a lung, followed by skin and soft tissue caused by rapid growers, such as MABSC or *M. gordonae*.

**Table 2:**
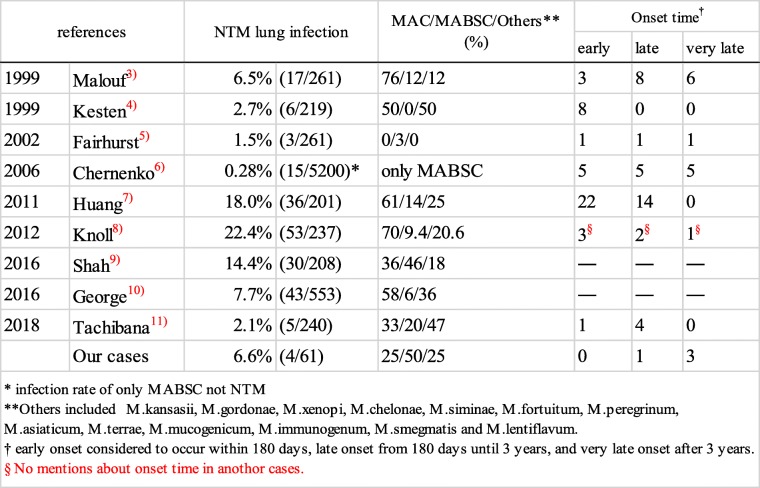
Previous reports of NTM lung infection after lung transplant

**Table 3:** Pathogens classified by onset time

	Early	Late	Very late
	(*n* = 43)	(*n* = 35)	(*n* = 16)
MAC^a^	4	13	5
MABSC^b^	11	10	9
M.gordonae	1	0	1
M.chelonae	1	0	0
M.siminae	0	1	0
M.kansasii	0	1	0
M.fortuitum	0	3	0
M.asiaticum	0	0	1
Not reported	26	7	0

^a^*MAC* mycobacterium avium complex

^b^*MABSC* Mycobacterium abscessus complex

We reviewed previous reports and classified those cases based on onset time after transplantation, with early onset considered to occur within 180 days, late onset from 180 days until 3 years, and very late onset after 3 years. There were 212 cases in which onset time was described (early onset, *n* = 43; late onset, *n* = 35; very late onset, *n* = 16) (Table 2), including 61 with both types of pathogens and onset time details (Table 3). MAC and MABSC were common organisms regardless of time of onset. In cases of early onset, the origin of NTM was newly acquired in some and prior to transplantation or donor-derived in others. On the other hand, patients in the very late onset group acquired a new infection under an immunosuppressed condition. All 16 cases had respiratory symptoms due to NTM pulmonary disease (Table 4). The average time from transplantation to onset was 6.1 years, and prognosis in most was good (Table 4). The direct cause of the three mortalities was not NTM, but rather bronchiolitis obliterans syndrome (BOS) in one [3], and either aspergillus or pneumonia infection in two [6].

**Table 4:** Details of very late onset reported cases

0	References		Age	Sex	Reason fot LuTx	LuTx	Time to onset	Symptoms	Chest X-ray findings	Culture	Pathogen	Drug for treatment	Outcome
1	1999	Malouf [3]	55	F	PAH	HL	8.9 years	Cough, sputum, dyspnea, fever	Normal	Sputum	MABSC	Ri + C + Ci	Alive
2			38	M	CHD	HL	4.3 years	Cough, sputum, dyspnea, fever	Bilateral apical infiltrate	BAL	M.avium	R + E + C + Ci	Alive
3			45	F	Empysema	SL	3.6 years	Cough, sputum, dyspnea, fever	Basal infiltrates	BAL	MAC	Ri + C + Ci	Alive
4			32	M	AATD	SL	3.6 years	Cough, dyspnea, fever	Apical and mid-zone infiltrates	Sputum	MAC	Ri + C + Ci	Dead
5			54	M	Empysema	SL	3.7 years	Cough, sputum, dyspnea, fever	Consolidation in midzone	BAL	M.avium	No treatment	Alive
6			48	M	AATD	SL	3.3 years	Cough, sputum, dyspnea, fever	Infiltrate in transpranted lung	Pleural fliud	M.asiaticum	R + E + P	Alive
7	2002	Fairhurst [5]	55	F	PAH	HL	9.0 years	Cough, sputum, dyspnea, fever	–	BAL	MABSC	Ri + C + Ci	Alive
8	2006	Chernenko [6]	40	F	Eisenmenger	HL	9.1 years	Fever, chills	–	Sputum	MABSC	R + C + Ci	Dead
9			48	M	COPD	SL	8.4 years	Fever, chills	–	BAL + sputum	MABSC	R + E + C	Dead
10			53	M	COPD	BL	8.5 years	Sputum	Normal	BAL + sputum	MABSC	R + E + Ci	Alive
11			39	M	CF	BL	9.3 years	Sputum	Nodules	Sputum	MABSC	Ri + C + Ci + E	Alive
12			42	F	LAM	BL	4.1 years	Sputum	Nodules	Sputum	MABSC	AMK + AZ + cefoxitin+Ci	Alive
13	2012	Knoll [8]	28	F	CF	BL	3.2 years	–	–	BAL	M.avium	–	Alive
14	2018	Our study	10	M	PAH	LDLT	6.3 years	None	Apical infiltrates	BAL	M.godnae	no treatment	Alive
15			35	F	BE	LDLT	6.8 years	Fever, sputum	Basal infiltrates	BAL	MABSC	TAZ/PIPC+AZM	Alive
16			41	F	LAM	SL	4.8 years	Sputum	Basal infiltrates	Sputum	MABSC	AZM + IPM + AMK	Alive

*AATD* α 1-antitrypsin deficiency, *AMK* amikacin, *AZM* azithromycin, *BAL* bronchoalveolar lavage, *BE* bronchiectasis, *BL* bilatetal lung, *C* Clarithromycin, CF cystic fibrosis, *CHD* congenital heart disease, *Ci* ciprofloxacin, *COPD* chronic obstructive pulmonary disease, *E* Ethambutol, *HL* heart-lung, *IPM* imipenem, *LAM* lymphangioleiomyomatosis, *LDLT* living donor lung transplantation, *P* Pyridoxal Phosphate Hydrate, *PAH* pulmonary arterial hypertension, *R* rifampicin, *Ri* Rifabutin, *SL* single lung, *TAZ/PIPC* Tazobactam / Piperacillin

Chronic rejection is known to be associated with NTM infection [3], and bronchiolitis obliterans syndrome is likely to occur in patients with NTM detected [7, 9]. Most of the patients in our cohort had respiratory symptoms, such as cough, sputum, and fever, with imaging showing consolidation suggesting pneumonia, and were diagnosed based on sputum culture or BAL results. Aggravation of a pulmonary infection in cases affected by immunosuppression will occur if an inappropriate antibiotic medicine is administered. Sputum obtained by expectoration may not be representative of sputum from deeper parts; thus, it is useful to first perform BAL to detect pathogens with the possibility of a combined infection of acid-fast bacteria or fungus kept in mind. The percentage of patients with MABSC was high (56.3%), which included therapy resistant cases ending in death from respiratory failure, though that rate may be falsely high because of selection bias. It is important for physicians to be aware that the risk of NTM infection continues for a long period after transplantation, as that was previously reported to occur in a patient after 9 years [6], and such occurrence may be via the same mechanism seen in the general population. NTM can destroy the lung structure, leading to decreased respiratory function [12]. Thus, care must be taken regarding reduction of immunosuppressive agents because respiratory function can worsen if rejection occurs. In most reports, overall survival after lung transplantation was not influenced by NTM infection [8, 9], though George et al. found that the survival rate of pulmonary NTM disease was lower than that of subclinical infection cases [10]. On the other hand, another investigation found that it was similar between cases of single lung transplantation and those with BOS [7].

Selection of the optimum antibiotic medication for treatment is crucial, though dependent on the sensitivity of each pathogen according to the ATS guidelines and must also be adjusted in accordance with the prescribed immunosuppressive agents. On the other hand, that is dependent on individual patient state regarding which agents can be reduced. PSL was decreased in two of our cases and only case 1 showed improvement with treatment. RFP, administered for MAC, attenuates the effect of ciclosporin; thus, it is necessary to increase its dose by 3–5 times above the standard when administered. The present case 4 was given an approximately threefold greater dose of ciclosporin while receiving the standard RFP dosage. Ciclosporin must be adjusted by monitoring its concentration in blood, as that varies in each patient from a variety of factors.

## Conclusions

An NTM infection and pulmonary disease can occur several years following lung transplantation. It is necessary to consider the best treatment for affected patients based on the pathogens involved, individual status, and disease severity, including which antibiotic medicine should be selected and which immunosuppressive agent can be decreased.

## Abbreviations

AATD: α-1 antitrypsin deficiency
AMK: Amikacin
AZM: Azithromycin
BAL: Broncho-alveolar lavage
BE: Bronchiectasis
BL: Bilatetal lung
BOS: Bronchiolitis obliterans syndrome
C: Clarithromycin
CF: Cystic fibrosis
CHD: Congenital heart disease
Ci: Ciprofloxacin
COPD: Chronic obstructive pulmonary disease
CT: Computed tomography
CyA: Ciclosporin
E: Ethambutol
HL: Heart-lung
IPM: Imipenem
LAM: Lymphangioleiomyomatosis
LDLT: Living donor lung transplantation
M.: Mycobacterium
MABSC: Mycobacterium abscessus
MAC: Mycobacterium avium complex
MMF: Mycophenolate mofetil
NSIP: Nonspecific interstitial pneumonia
NTM: Nontuberculous mycobacteriosis
P: Pyridoxal phosphate hydrate
PAH: Pulmonary arterial hypertension
PSL: Prednisolone
R: Rifampicin
REC: Rifampicin, ethambutol, and clarithromycin
RFP: Rifampicin
Ri: Rifabutin
SL: Single lung
TAZ/PIPC: Tazobactam/piperacillin
